# Unusual crystal structures of MHC class I complexes reveal the elusive intermediate conformations explored during peptide editing in antigen presentation

**DOI:** 10.21203/rs.3.rs-2500847/v1

**Published:** 2023-01-26

**Authors:** Lenong Li, Xubiao Peng, Mansoor Batliwala, Marlene Bouvier

**Affiliations:** 1 Department of Microbiology and Immunology, University of Illinois, Chicago, IL, 60612, USA; 2 Center for Quantum Technology Research and Key Laboratory of Advanced Optoelectronic Quantum Architecture and Measurements (MOE), School of Physics, Beijing Institute of Technology, Beijing 100081, China

## Abstract

Studies have suggested that MHC class I (MHC I) molecules fluctuate rapidly between conformational states as they sample peptides for potential ligands. To date, MHC I intermediates are largely uncharacterized experimentally and remain elusive. We present x-ray crystal structures of HLA-B8 loaded with 20mer peptides that show significant conformational heterogeneity at the N-terminus of the groove. Long stretches of N-terminal residues were missing in the electron density maps creating an unstructured and widely open-ended groove. Our structures also revealed highly unusual features in MHC I and peptide conformations, and in MHC I-peptide interaction at the N-terminus of the groove. Molecular dynamics simulations showed that the complexes have varying degrees of flexibility in a manner consistent with the structures. We suggest that our structures represent transient substates explored by MHC I molecules during peptide editing. The visualization of peptide-dependent conformational flexibility in MHC I groove is a major step forward in our conceptual understanding of peptide repertoire development in antigen presentation. Our study also raises questions about the role of the N-terminus of the groove in peptide editing.

## Introduction

Antigen presentation by major histocompatibility class I (MHC I) molecules is central to adaptive immunity. MHC I molecules bind peptides and present them at the cell surface to specific receptors on CD8+ T cells. This surveillance alerts the immune system to the presence of virally infected and transformed cells. MHC I molecules binds peptides in a groove that is lined with discrete pockets (A to F)^1^. The stability of MHC class I molecules is highly dependent on interaction with a bound peptide ligand^2–5^. As such, within the endoplasmic reticulum (ER), there is an elaborate network of specialized proteins, referred to as the peptide-loading complex (PLC)^6^, that ensures MHC I molecules are loaded with high-affinity peptides prior to their transport to the cell surface. Studies of the mechanism by which high-affinity peptides become ligands of MHC I have highlighted that the F pocket at the C-terminal end of the groove is a critical region of conformational sensing^7–15^. Indeed, the PLC proteins tapasin, ERp57, and calreticulin are spatially organized on MHC I molecules using sites of interaction at the C-terminal end of the groove^16^.

Molecular dynamic (MD) studies have suggested that the MHC I groove fluctuates rapidly between conformations, and it is the molecular features of such intermediate states that are recognized by specialized proteins, particularly tapasin^7–15^. The binding of tapasin to immature MHC I molecules induces a widening of the groove thereby encouraging the dissociation of non-optimally bound peptides^17^. Ultimately, under the action of tapasin, MHC I peptide repertoires are largely made up of highly stabilizing peptides, which ensures long-lived antigen presentation at the cell surface. An understanding of the molecular mechanism by which tapasin functions has been enriched from studies of TAPBPR^18–22^, a tapasin homolog that works independently of the PLC. Although there is direct evidence that dynamics in MHC I play a fundamental role in mechanisms of peptide selection and exchange^23^, the molecular features of MHC I intermediate states at the core of these processes are elusive. Except for some information on the peptide-receptive form of MHC I^24–27^, conformational intermediates of MHC I-peptide complexes are largely undefined due to the difficulty of probing such transient molecules. Furthermore, it is not well characterized if the region around the critical A and B pockets, at the N-terminal end of the groove, represents another site of conformational sensing and if it has a role in peptide editing.

In previous studies, we characterized crystallographically the presentation of long peptides based on the sequence (RA)_n_AAKKKYCL by HLA-B8E76C^28,29^. We showed that these peptides adopt native bound conformations with their N-terminally elongated residues (RA)_n_ protruding out of the groove. These structures provide a platform for the rationale design of peptides that have the potential to induce conformational perturbations at the N-terminal end of the groove and, therefore, that could provide direct insights into non-native MHC I conformations. Toward this goal, we substituted Ala at position 1 (P1) and P2 in (RA)_6_AAKKKYCL 20mer peptide with the bulkier residues Phe and Val generating (RA)_6_FAKKKYCL and (RA)_6_FVKKKYCL. We present the x-ray crystal structures of HLA-B8 loaded with these long peptides that show highly unusual features in MHC I and peptide conformations and in MHC I-peptide interaction at the N-terminus of the groove. Our structures reveal for the first-time motions that MHC I molecules likely undergo transiently during the dynamic steps of peptide sampling. We combined these structural analyses with MD simulations which revealed other aspects of MHC I-peptide interaction in our complexes that are relevant for peptide editing, and consistent with the structures. Overall, our study provides a precise understanding of the link between molecular dynamics in MHC I molecules and their ability to adopt intermediate conformations for screening peptides efficiently, which is fundamentally important to ensure effective cytotoxic T cell responses to viral infections.

## Results

### Strategically designed peptides

We designed two HLA-B8-restricted 20mer peptides based on the sequence of HIV-1 Gag epitope GGKKKYKL^30^ in which P1 was substituted with Phe, P2 with either Ala or Val, and P7 with Cys. The resulting FAKKKYCL and FVKKKYCL peptides were N-terminally extended with twelve residues (RA)_6_ generating (RA)_6_FAKKKYCL and (RA)_6_FVKKKYCL. The P7 Cys was introduced to form a disulfide bond with Cys76 in HLA-B8, after mutating Glu76, to prevent the dissociation of long peptides from the groove^28,29^. The 8mer FAKKKYCL and FVKKKYCL control peptides were also synthesized. The reconstitution of HLA-B8E76C-peptide complexes was carried out *in vitro* as we described previously^28,29^.

### Unconventional peptide binding and presentation

**Figure 1.**
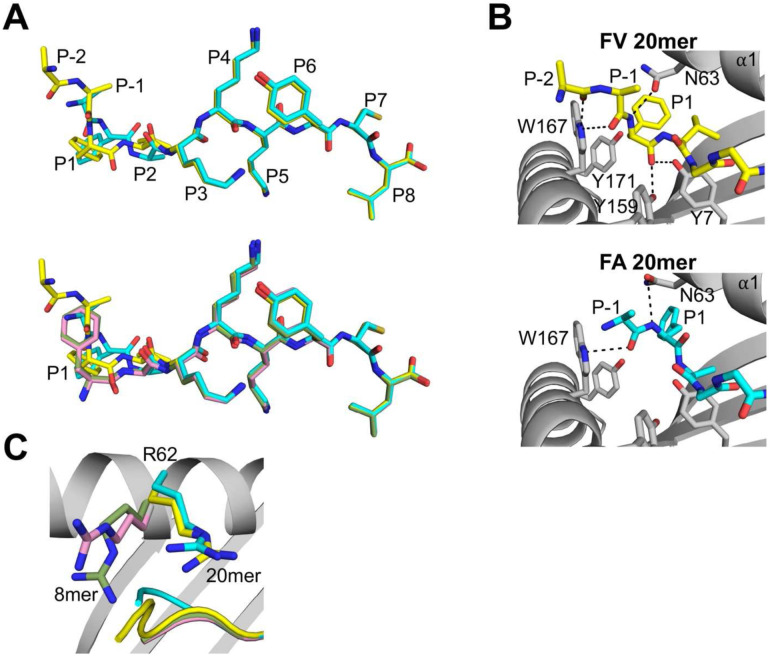
Unusual binding and presentation of (RA)_6_FAKKKYCL and (RA)_6_FVKKKYCL. **A**, Top panel, superimposition of bound (RA)_6_FAKKKYCL (cyan) and (RA)_6_FVKKKYCL (yellow) 20mer peptides. The backbone and side chain conformations of the peptides overlap between P3 and P8 but differ at P1, P2, and P-1 (P-2 was visible only in 20mer FV). Bottom panel, superimposition of bound 20mer peptides with 8mer FAKKKYCL (pink) and FVKKKYCL (green) control peptides. The four peptides overlap between P3 and P8 and are most divergent at P1. **B**, Interactions in the A pocket show that the main-chain nitrogen of P1 Phe FV 20mer (top panel) and FA 20mer (bottom panel) has rotated and forms a hydrogen bond with Asn63 (black dashed lines). The main-chain carbonyl oxygen in FV 20mer hydrogen bonds with Tyr159 and Tyr7, while the same atom in FA 20mer has undergone a very unusual rotation toward the α1-helix and forms no interaction with MHC I residues. In both panels, extension residues protrude out of the groove. **C**, In the 20mer structures, the side chains of Arg62 have moved out of the canonical positions seen in the 8mer structures, which opens the A pocket and allows the extension residues (RA)_6_ to exit out.

We determined the x-ray crystal structures of HLA-B8E76C loaded with the 20mer and 8mer peptides to high-resolution (Supplementary Table 1). The structures show that FA (cyan) and FV (yellow) 20mers adopt elongated conformations in which the core residues P1 to P8 are bound inside the groove and the extension residues P-1 Ala (one position N-terminal to P1) and P-2 Arg (visible only for FV 20mer) protrude out of the groove (Fig 1A, upper panel). The peptide backbones and side chains adopt nearly identical positions between P3 and P8, but clear differences are seen at P-1, P1, and P2. The Cα-atom positions have a 2.84-Å shift at P1 and 0.96-Å shift at P2. Comparisons of FA and FV 20mers with their corresponding FA (pink) and FV (green) 8mers (Fig 1A, lower panel) show that 8mer peptides adopt nearly identical bound conformations, and that P1 is the most divergent position in both 20/8mer pairs, with shifts in Cα-atom of 3.13-Å for FAs and 1.42-Å for FVs. Finally, the electron density was clear over the entire length of the peptides, including the backbone and methyl side chain of P-1 Ala and the backbone and part of the aliphatic side chain of P-2 Arg (FV 20mer only) (Supplementary Fig. 1).

A close examination of how the FV 20mer peptide binds in the A pocket (Fig 1B, upper panel) shows that the main-chain nitrogen of P1 Phe is rotated in a position that is normally occupied by a canonical P1 side chain, as seen in FA and FV 8mers (Fig. 1A, lower panel). In this configuration, the P1 main-chain nitrogen forms a hydrogen bond with Asn63, and the extension residues P-1 Ala and P-2 Arg protrude out of the A pocket. Interestingly, the main-chain carbonyl oxygens of P-1 and P-2 residues form hydrogen bonds with the indole nitrogen of Trp167 (Fig 1B, upper panel), which likely stabilize the peptide backbone as it exits out of the groove. The structure also shows that the bulky P1 Phe side chain cannot occupy the terminal amino group canonical position, i.e., the cavity formed by residues Tyr7 and Tyr171^1^, and instead the phenyl ring points toward the α1-helix (see also Fig. 4A, right panel). Finally, the main-chain carbonyl oxygen of P1 Phe forms hydrogen bonds with Tyr7 and Tyr159 (Fig 1B, upper panel). A similar rotation within the A pocket was also seen in the FA 20mer structure (Fig 1B, lower panel), with P1 main-chain nitrogen and P-1 carbonyl oxygen forming hydrogen bonds to Asn63 and Trp167, respectively. In marked contrast to FV 20mer, however, the main-chain carbonyl group of P1 Phe is rotated toward the α1-helix, a highly unusual configuration, and surprisingly does not engage with any MHC I residues (see also Fig. 4A, left panel). Taken together, although P1 Phe residues of 20mer peptides have undergone a similar main-chain nitrogen rotation in the A pocket, there are clear differences in the binding mode of each peptide. Finally, a comparison of Arg62 in FA and FV 20mer structures relative to the 8mer structures (Fig. 1C) shows that the Arg side chains swing out of their canonical positions which creates an opening for (RA)_6_ residues to exit out of the groove. A similar role of residue 62 in opening the A pocket was observed in our structure of (RA)_6_AAKKKYCL 20mer peptide bound to HLA-B8E76C^29^.

### Significant peptide-induced conformational distortions at the N-terminus of the groove

**Figure 2.**
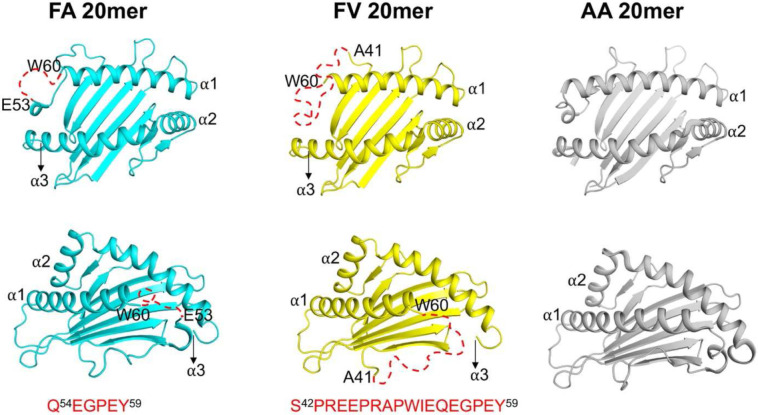
Top and side views of HLA-B8E76C groove in FA, FV, and AA 20mer structures. The figure shows peptide-induced structural distortions at the N-terminus of the groove. Residues Gln54 to Tyr59 (6 residues) and Ser42 to Tyr59 (18 residues) are not visible (shown as red dashed lines) in the FA and FV 20mer structures, respectively. This is in marked contrast to AA 20mer structure which has a correctly conformed groove^29^.

The binding modes of FA and FV 20mers described in Fig. 1 are accompanied with significant structural changes in the MHC I groove. Figure 2 shows top and side views of the MHC I groove of FA and FV 20mer structures in which long stretches of N-terminal residues (shown by dashed red lines) in the α1-helix and loop connecting the α1-helix to β-strand of the floor were missing in the electron density maps. Specifically, the FA 20mer structure lacks 6 residues from Gln54 to Tyr59, and FV 20mer structure lacks as many as 18 residues from Ser42 to Tyr59 - electron densities at an acceptable 1σ threshold were not visible for these residues in our structures. Consequently, the A pocket is unstructured and widely open-ended (Fig. 2 and Supplementary Fig. 2). In contrast, our previously determined AA 20mer structure showed that the N-terminus of the groove has a native structure (Fig. 2)^29^. Thus, the FA and FV 20mer structures provide direct evidence that the N-terminus of the groove has the potential to undergo significant peptide-induced structural distortions, highlighting its remarkable inherent plasticity. To the best of our knowledge, this is the first report of MHC I structures, with or without a bound peptide, showing such significant conformational distortions in the groove. Other than these differences, minor changes in the groove were detected between the FA and FV 20mer structures (r.m.s. deviation of 0.13-Å). It is interesting that FA and FV 20mer peptides differ only by the nature of residues at P1 and P2 relative to our previous AA 20mer peptide^29^. Because all N-terminal MHC I residues were visible in the AA 20mer structure (Fig. 2), but not in the FA and FV 20mer structures, this strongly suggests that P1 and P2 residues play critical roles in the conformational maturation of the groove.

### Unusual MHC I-peptide interaction at the N-terminus of the groove

**Figure 3.**
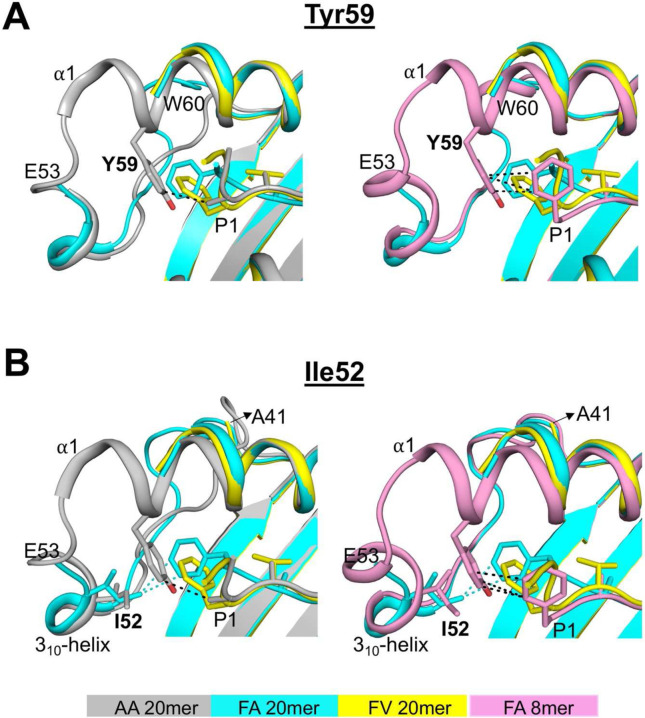
Key roles of N-terminal MHC I residues Tyr59 and Ile52. **A**, Superimposition of FA and FV 20mer structures with AA 20mer (left panel) and FA 8mer (right panel) structures. The P1 Ala side chain of AA 20mer and P1 Phe side chain of FA 8mer occupy canonical positions and interact with Tyr59 (black dashed lines), while the bulky P1 Phe side chains of FA and FV 20mers clash with Tyr59 causing conformational disorders between Gln54 to Tyr59 (left and right panels). **B**, Same superimpositions as in **A**. The P1 Phe side chain of FA 20mer interacts with Ile52 of the 3_10_-helix (cyan dashed lines) which stabilizes Ser42 to Tyr59 (left and right panels), while a similar interaction involving Ile52 is not possible for FV 20mer resulting in conformational disorders between Ser42 to Tyr59 (left and right panels). The P1 Ala side chain of AA 20mer (left panel) and P1 Phe side chain of FA 8mer (right panel) have no interaction with Ile52.

To understand the role of peptide P1 residue in modulating interactions with MHC I residues at the N-terminus of the groove, we analyzed the FA and FV 20mer structures in the context of both the AA 20mer^29^ and FA 8mer structures (Fig. 3). The structure of AA 20mer shows that the small P1 Ala methyl side chain forms a hydrophobic interaction with conserved residue Tyr59 (Fig. 3A, left panel). In contrast, the large P1 Phe side chains of FA and FV 20mers sterically clash with Tyr59 (Fig. 3A, left panel) and as such, residues Gln54 to Tyr59 become disorganized and are not visible in the FA and FV 20mer structures while these residues are conformed in AA 20mer structure. Interestingly, the native structure of FA 8mer (Fig. 3A, right panel) shows that the P1 side-chain phenyl ring occupies a canonical position and forms hydrophobic interactions with Tyr59, and that residues Gln54 to Tyr59 are visible. Similar observations were made in the FV 8mer structure (Supplementary Fig. 3). Taken together, our structures indicate that residues Gln54 to Tyr59, and especially Tyr59, form a region of remarkable conformational flexibility and structural plasticity that allows adaptations in response to size and configuration of peptide P1 side chains.

There is another unusual feature at the N-terminus of the groove in FA 20mer structure (Fig. 3B). In this structure (Fig. 3B, left panel), the P1 side-chain phenyl ring forms highly unusual hydrophobic interactions with the highly conserved Ile52 of the 3_10_-helix (a conserved element comprising residues 50 to 55). In the FV 20mer structure, however, the P1 phenyl ring is oriented differently (Fig. 3B, left panel) and, as such, it cannot engage with Ile52 thus causing residues Ser42 to Glu53 to become disordered. In the FA20mer structure, residues Ser42 to Glu53 were clearly visible due to P1 phenyl ring engagement with Ile52. Notably, interaction between a bound peptide and Ile52, or any other residues of the 3_10_-helix, have not been reported before and are also not seen in FA and FV 8mer structures (Fig. 3B, right panel, and Supplementary Fig. 3). Taken together, there are clear molecular interplays involving peptide P1 residue and N-terminal residues Tyr59 and Ile52 that influence structural integrity at the N-terminal end of the groove (see also below).

### Molecular cross-talks between peptide P1 and P2 residues and N-terminal MHC I residues Ile52 and Tyr59

**Figure 4.**
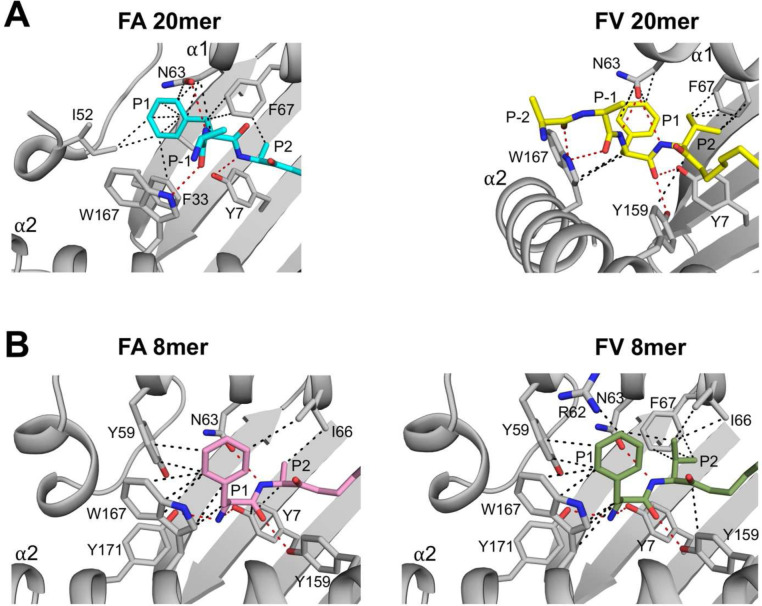
Molecular cross-talks between occupied pockets A and B. **A**, P1 phenyl side chain is oriented differently in FA 20mer (left panel) relative to FV 20mer (right panel). This is due to molecular cross-talks between peptide P1 and P2 residues and Ile152 and Tyr59 (missing) (see text and Fig. 3B). Overall, the network of hydrophobic (black dashed lines) and hydrogen bond (red dashed lines) interactions in the A and B pockets are different in these two structures. **B**, The network of interactions in FA 8mer (left panel) and FV 8mer (right panel) is quite similar overall in these conformed structures, in contrast to FA and FV 20mer structures shown in A.

Results in Fig. 3 raise another important question: why is the P1 Phe side chain oriented differently in FA 20mer versus FV 20mer structures? This question is important given that this difference in orientation significantly affected the structural integrity of Ser42 to Glu53 in the FV 20mer structure. To address this, we examined peptide P2 residues, which is Ala in FA 20mer and Val in FV 20mer (Fig. 4A). In the FA 20mer structure (Fig. 4A, left panel), the small side-chain methyl group of P2 Ala allows the P1 main-chain carbonyl group to undergo an unusual rotation toward the α1-helix, which has the effect of orienting the P1 phenyl ring close to Ile52. In the FV 20mer structure, however, because P2 carries a larger Val side chain (Fig. 4A, right panel), the P1 main-chain carbonyl group cannot similarly rotate toward the α1-helix, and consequently the P1 phenyl ring is positioned further away from Ile52. Overall, different networks of interactions involving peptide P1 and P2 residues and N-terminal MHC I residues were established in FA and FV 20mer structures (Fig. 4A). Interestingly, using a thermal denaturation assay, we determined rather similar melting temperature (Tm), 65.8°C for FA 20mer and 67.5°C for FV 20mer. In contrast, the structures of FA and FV 8mer show very similar networks of interactions (Fig. 4B) and identical Tm values, 71.1°C for FA 8mer and 71.4°C for FV 8mer.

### Analysis of MHC I residues in pockets along the groove

Given the importance of the six binding pockets A to F in determining the peptide side chain specificities of HLA alleles, we compared the side chain orientations of MHC I residues in pockets A and B of FA and FV 20mer structures relative to the corresponding 8mer structures (Supplementary Fig. 4). Pocket A is made up of 9 residues and typically anchors the N-terminal amino group and P1 residue and closes the N-terminal end of the groove. The analysis shows that Tyr59 (conserved) and Asn63 (highly conserved) have the most divergent orientations in both FA and FV 20/8mer pairs, with some differences also seen in Tyr171 (conserved) and Trp167 (highly conserved). Pocket B is made up of 9 residues and binds the P2 peptide side chain that defines HLA binding motifs. For both the FA and FV 20/8mer pairs, all MHC I residues adopted very similar orientations, except for Asn63 at the boundary of the A and B pockets. Similar analyses in pockets C to F indicated that there were minimal changes in MHC I side chain orientations (data not shown). Taken together, the binding of 20mer peptides affects the configuration of MHC I residues in pocket A more significantly than those in pocket B, consistent with P1 being the most divergent position of these peptides (Fig. 1A). This analysis also highlighted a critical role for Asn63 in MHC I maturation; while Asn63 mediated several hydrophobic and hydrogen bond interactions with P1 and P2 residues in the FA and FV 20mer structures (Fig. 4A), Asn63 formed only one hydrogen bond with P2 main-chain nitrogen in the native FA and FV 8mer structures (Fig. 4B).

### MD simulations: conformational flexibility and geometrical parameters

To further characterize HLA-B8E76C-peptide complexes and interaction between peptide P1 residue and Tyr59 and Ile52, we analyzed thermal properties, i.e., conformational flexibility and geometrical parameters, from MD simulations at physiological temperature.

**Figure 5.**
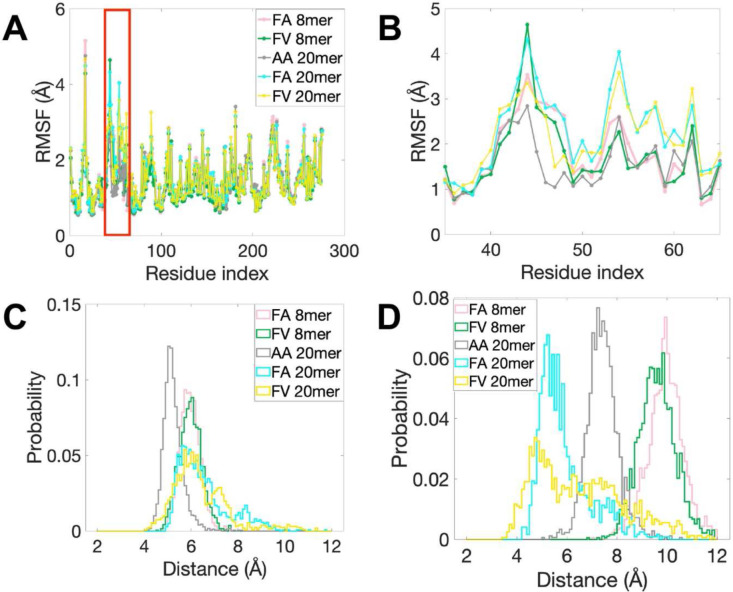
Properties of equilibrium ensembles of HLA-B8E76C-peptide complexes. **A**, RMSF values of individual residues along the heavy chain of HLA-B8E76C loaded with different peptides highlighting that the highest values are in the region of residues 41 to 62 (shown in a red box). **B**, A zoom-in of panel **A** reveals two distinct regions, residues 41 to 46 (peptide-independent) and residues 52 to 62 (peptide-dependent). **C**, Probability distributions of inter-residue distance between peptide P1 and Tyr59 and **D**, peptide P1 and Ile52 for HLA-B8E76C loaded with different peptides (see text).

The flexibility of individual MHC I residues along the heavy chain is characterized by its root mean square fluctuation (RMSF) (Fig. 5A). Results show that RMSF values are clearly higher in the region comprising residues 41 to 62 (shown in a red box) for FA and FV 20mer complexes relative to other regions, indicating that residues 41 to 62 are conformationally more flexible and thermally more unstable. A zoom-in revealed that there are two distinct regions (Fig. 5B); residues 41 to 46 and residues 52 to 62. In the region of residues 52 to 62, the RMSF values for FA and FV 20mer complexes are much higher than those of the other complexes, indicating that the 20mer complexes are conformationally more flexible in this region. However, in the region of residues 41 to 46, all complexes have similarly high RMSF values, suggesting that conformational flexibility is independent on the bound peptide in this region.

We evaluated next the geometrical parameters of interaction between peptide P1 residue and Tyr59 and Ile52 to gain further insights into these complexes (see Methods for details). Figure 5C shows the probability distributions of inter-residue distance between P1 and Tyr59 for all five systems. Results show that the peak of the distribution is centered around 5Å for AA 20mer while the other distributions are centered around 6Å, suggesting a high probability of interaction between P1 and Tyr59 in all complexes when equilibrated at physiological temperature. However, because the distributions are broader for FA and FV 20mers than for the other systems, this suggests that interaction between P1 and Tyr59 is less stable in the 20mer complexes. We also calculated the probability distributions of relative orientation between the aromatic rings of P1 Phe and Tyr59 (Supplementary Fig. 5A). Results show that FA and FV 8mers have symmetric distributions centered around 90°, indicating that the two aromatic rings are essentially perpendicular to each other. In contrast, for FA and FV 20mers, the peaks of the distributions are positioned at 110° and 150°, respectively, showing that the aromatic rings of P1 Phe and Tyr59 are no longer perpendicular to each other. Accordingly, we conclude that the configuration of π-π interactions^31^ between P1 Phe and Tyr59 is more parallel-like in FV 20mer but changes toward T-shape-like in FA 20mer, and finally adopts the stable T-shaped structure in FA and FV 8mers. Finally, Fig. 5D clearly shows that the probability distributions of inter-residue distance between P1 and Ile52 are different among the five complexes. For FA 20mer, the distribution has a high peak centered around 5Å, indicating that there is a stable interaction between these two residues. For FV 20mer, the distribution still peaked around 5Å but it is much wider, indicating that interaction between P1 and Ile52 is less stable (Fig. 5D). In contrast, the distributions for FA and FV 8mers have high peaks centered around 10Å, indicating that there are no interactions between P1 and Ile52 in these complexes. For AA 20mer, since the distances are centered around 7Å, we infer that weak hydrophobic interaction between P1 and Ile52 may exist in this complex. To further characterize interaction between P1 and Ile52 in FA and FV 20mers, we determined the probability distributions of angle C-H-X (X being the center of Phe aromatic ring) (Supplementary Fig. 5B). Results show that the angles are mostly in the range 120° to 150°. As such, since the probability distributions of “distance” (Fig. 5D) and “defined angle” (Supplementary Fig. 5B) between P1 and Ile52 satisfy the geometric criterion of CH-π interaction^32^, we conclude that CH-π interaction exists in both FA and FV 20mers.

### MD simulations: rotation of peptide terminal amino group in the A pocket

Given that the terminal amino groups of FA and FV 20mers adopt highly unusual configurations in the A pocket (Fig. 1B), we conducted MD simulations to assess whether these amino groups can undergo spontaneous rotations to canonical positions, i.e., pointing down in the A pocket. For these tests, we removed the extension (RA)_6_ residues of FA and FV 20mer peptides in their structures, generating FA20..8mer and FV20..8mer (see Methods for details). The definition of the dihedral angle ω for characterizing the rotation of the terminal amino group is shown in Fig. S6A, using as an example FV20..8mer. In the FA and FV 20mer structures, ω values are −110° and −73°, respectively, indicating that both terminal amino groups point up, while in the native FA and FV 8mer structures, ω values are 96° and 98°, respectively, indicating that the amino groups point down.

The evolution curves of the dihedral angle ω in MD simulations for FA20..8mer and FV20..8mer are shown in Supplementary Figs. 6B and 6C, respectively. For FA20..8mer, the results show that ω changes from about −100° to 100° in two out of the three replicate simulations within 300 ns. For FV20..8mer, ω changes from about −70° to around −260° in two out of the three replicate simulations within 300 ns (note that ω = −260° is equivalent to ω = 100°, when the periodicity of ω is taken into consideration). Hence, we conclude that the terminal amino groups of FA20..8mer and FV20..8mer have high probability to rotate in the A pocket and point down as seen in native structures. In addition, it is interesting that different replicas gave different evolution curves of the dihedral angle ω, indicating that the pathways of such rotations are unlikely to be unique.

### Analysis of our structures in the context of bat MHC I molecules and human MHC II molecules

**Figure 6.**
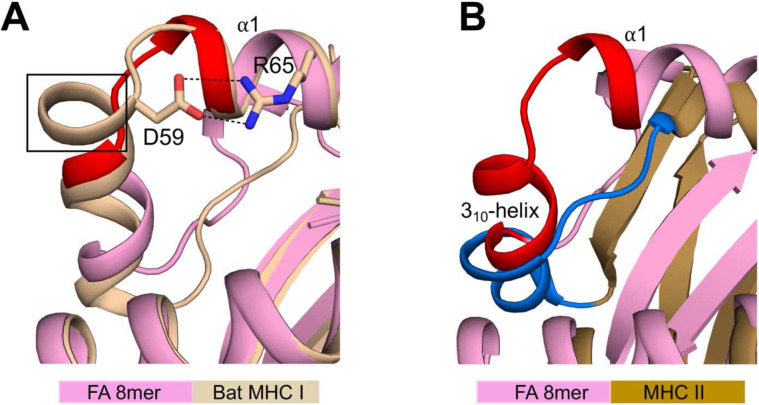
Comparisons with the structures of bat MHC I and human MHC II molecules. **A**, Superimposition of FA 8mer and bat MHC I molecule (Ptal-N*01:01; PDB code 6J2D) structures. The bat molecule has an additional turn (highlighted by a black box) in the extended region of Gln54 to Tyr59 (highlighted in red) that we identified as disordered (see Fig. 2). In this turn, Asp69 forms salt bridge interactions (black dashed lines) with Arg65 of the α1-helix. **B**, Superimposition of FA 8mer and human MHC II molecule (HLA-DR1; PDB code 1DLH) structures showing that the HLA-DM susceptible region, i.e., 3_10_-helix and unstructured loop (highlighted in dark blue) overlaps with the 3_10_-helix and extended region (highlighted in red) that we identified as critical in shaping the A and B pockets (see Fig. 4).

The characterization of bat MHC I genes identified thus far, showed that many of these molecules contain a 3- or 5-amino acid insertion in their binding groove^33^. Several structures of Ptal-N*01:01 bat MHC I molecules with a 3-amino acid insertion have been determined and revealed that the insertion creates a turn at the N-terminus of the groove (Fig. 6A)^34,35^, within the critical region of Gln54 to Tyr59 (shown in red). The bat structures also revealed that in this turn, Asp59 forms salt bride interactions with Arg65 of the α1-helix (Fig. 6A)^34,35^. Such a pairing of charged residues at these positions is a highly conserved feature of bat MHC I molecules^34,35^, and it is expected to add structural rigidity at the N-terminus of the groove relative to human MHC I molecules. As such, the groove of bat MHC I molecules may be more restricted in its ability to adopt alternate conformations, which could affect peptide editing and repertoire development with implication on bat adaptive immunity. Further investigation is required to understand these observations.

Finally, in MHC class II (MHC II) molecules, there is evidence of peptide-induced conformational flexibility at the N-terminus of the groove^36–38^, i.e., the region recognized by the peptide-exchange catalyst HLA-DM^39,40^. The binding of HLA-DM induces conformational changes in MHC II, particularly in the 3_10_-helix and unstructured loop^41^ (shown in dark blue in Fig. 6B). This critical region of MHC II overlaps with the region of MHC I (3_10_-helix and extended region) that we identified as important for shaping the A and B pockets (shown in red in Fig. 6B). This analysis lends support to the view that the N-terminal end of the groove likely has a role in peptide editing (see Discussion).

## Discussion

The crystal structures of native peptide-filled MHC I molecules have taught us a great deal about molecular recognition of bound peptides. These structures represent the endpoint of a complex intracellular maturation process whereby MHC I molecules acquire peptides of sufficiently high affinity to ensure efficient antigen presentation. Biophysical, NMR, and *in silico* studies have been consistent in demonstrating that MHC I molecules explore intermediate conformational states in solution during peptide binding. Given that the native crystal structures of peptide-filled MHC I molecules are always nearly identical, it has not been possible thus far to obtain detailed structural information of intermediate conformations explored by MHC I molecules. This makes the notion of functional dynamics and peptide-induced conformational motions elusive. We have determined the crystal structures of HLA-B8 loaded with 20mer peptides that reveal highly unusual conformational and structural features in both MHC I and peptides. We have also carried out MD simulations to gain further insights into peptide-dependent interaction in these complexes, which provided new information and helped refine our structural analyses.

**Figure 7.**
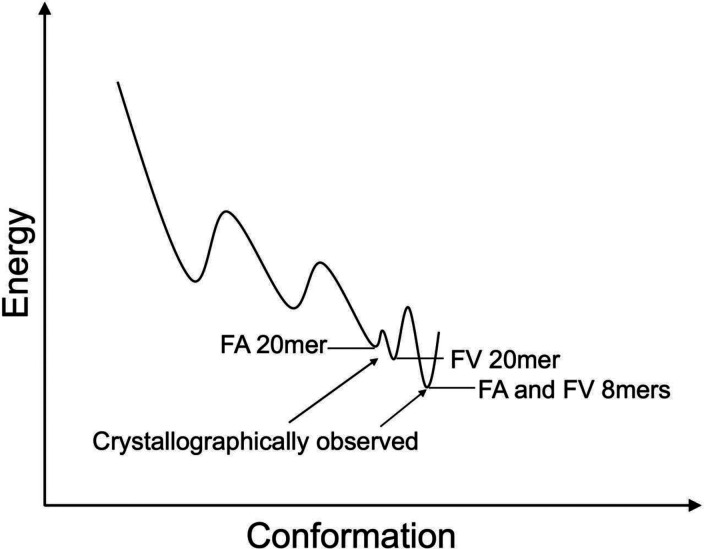
A model of energy landscape. The model depicts FA and FV 20mer complexes as intermediate states that interconvert and are of higher energy relative to the more conformed states seen in the structures of FA and FV 8mers.

The FA and FV 20mer structures showed that the N-terminus of the groove has undergone significant conformational distortions relative to native FA and FV 8mer structures (Fig. 2 and Supplementary Fig. 2). These differences were peptide specific. MD simulations identified that residues 52 to 62 are most conformationally flexible and thermally unstable in FA and FV 20mer complexes relative to the other complexes (Fig. 5B), consistent with the lack of electron density for residues 54 to 59 in FA and FV 20mer structures. MD simulations also identified a conformationally flexible region comprising residues 41 to 46 that seems more peptide independent. Consistent with this, it is interesting that several deposited structures of HLA-B8 loaded with 9mer peptides lack clear electron density between residues ~41 to 49 (for example, 1M05, 3SKO, 4QRQ, and 5WMR), suggesting that some conformational fluctuations can persist at the N-terminus of the groove in native structures. Our structures also showed that the 20mer peptides, with only a single amino acid difference at P2, adopted different and unusual backbone and side chain orientations at P1 and P2 but overlapped almost identically between P3 to P8 (Fig. 1A, upper panel). Interestingly, both complexes had similar thermostabilities, 65.8°C (FA 20mer) versus 67.5°C (FV 20mer). We suggest that the FA and FV 20mer structures, although static snapshots, represent discrete states that are explored by these complexes as they navigate the conformational space to locate their low energy conformation (“native”) (Fig. 7) (see below).

MHC I peptide ligands are usually 8 to 10 amino acids long and bind with their terminal amino group pointing down in the A pocket, as seen in the FA and FV 8mer structures (Fig. 4B). Because short linear peptides are generally unstructured in solution, it is reasonable to assume that they land as such within the immature MHC I groove. NMR and other biophysical studies showed that in the initial binding steps, incoming peptides are loosely accommodated in the groove until more specific conformational adaptions take place in both peptides and MHC I^22,23,42,43^. It is therefore plausible that when peptides of optimal lengths are first captured by MHC I, they adopt conformations that resemble those of our 20mer peptides, i.e., with an unusual rotation of the terminal amino group in the A pocket (Fig. 1B), until folding proceeds and peptides adopt a canonical conformation (or not). To test this, we simulated the relaxation process of FA20..8mer and FV20..8mer using plain MD simulations and the results showed that peptide terminal amino groups have a high probability to rotate to a canonical position from the unusual orientations observed in our structures. The simulations also suggested that the configurations of FA and FV 20mer peptides, as seen in the structures, represent “trapped” states (see below). Finally, it is worth noting that naturally occurring HLA-B8-restricted 8 and 9mer peptides with a large residue at P1 or at P1/P2 have been reported: for example, Phe, Val, Leu, and His at P1^44–47^ or Tyr/Leu, Trp/Val, Phe/Leu, and Tyr/Ile at P1/P2^44,48,49^.

The FA and FV 20mer structures showed that strictly conserved Tyr59 and highly conserved Ile52 (3_10_-helix) act synergistically at the N-terminus of the groove. In native MHC I structures, Tyr59 stabilizes the peptide terminal amino group together with Tyr171, and Ile52 acts as a structural support to Tyr59 and Tyr171^1^. In our 20mer structures, Tyr59 could not adopt its native position because of the large size of P1 Phe side chain in the A pocket (Fig. 3A). This caused residues 54 to 59 to become disordered and created an open-ended A pocket. In the FA 20mer structure, this disordering was accompanied with Ile52 forming a highly unusual interaction with peptide P1 Phe side chain, which in turn was facilitated by the small peptide P2 Ala side chain in the adjacent B pocket (Fig. 4A). In the FV 20mer structure, however, similar molecular cross-talks between the A and B pockets were not possible because of the larger peptide P2 Val side chain in the B pocket that positioned P1 Phe side chain further away from Ile52 (Fig. 4A). Consequently, a significantly longer stretch of residues became disordered in the FV 20mer structure, generating a widely open-ended A pocket. In performing MD simulations, we were able to probe other aspects of the interaction between peptide P1 and MHC I residues Tyr59 and Ile52 in the thermal equilibrium ensembles of our complexes. The simulations revealed that 1. interaction between P1 and Tyr59 is characterized by the more stable T-shaped π-π configuration in FA and FV 8mer complexes relative to FA and FV 20mer complexes (Fig. 5C and Supplementary Fig. 5A), which is consistent with residues 54–59 being ordered in the 8mer structures but disordered in the 20mer structures; and 2. CH-π interaction between P1 and Ile52 exists only in FA and FV 20mer complexes, and it is more stable in FA 20mer than FV 20mer (Fig. 5D and Supplementary Fig. 5B). This is also consistent with the FV 20mer structure showing a more highly disordered groove and widely open-ended A pocket. Taken together, we suggest that the 20mer structures represent “trapped” states, i.e., conformations in which Tyr59 cannot adopt a “closed” position (FA and FV 20mer structures) and 3_10_-helix cannot mature into its secondary fold (FV 20mer structure). In other words, formation of native A and B pockets requires the conformational transitions of Tyr59 into a “closed” position and 3_10_-helix (Ile52) into its native fold. A role for the 3_10_-helix in peptide-induced MHC I maturation was suggested previously^50–52^. Because Tyr59 and 3_10_-helix are strictly conserved elements in human HLA alleles, these transitions are expected to be universal in MHC I maturation. Furthermore, that the 20mer structures can tolerate such high degrees of peptide-induced structural perturbations at the N-terminus of the groove is consistent with MD simulations of other groups showing that bound peptides can dissociate partially at the N-terminus of groove while remaining anchored within the F pocket^53–55^, with potential implication in MHC I maturation (see below). Finally, the lower thermostabilities of FA and FV 20mer complexes relative to the 8mer complexes is consistent with the “trapped” states being higher energy conformations (Fig. 7), but of sufficiently low energy to be revealed crystallographically.

The conformational transitions discussed above are intricately coupled to occupancy of the A and B pockets. This is significant when considering that the crystal structure of a peptide-free MHC I molecule (HLA-A2) showed no substantial differences at the N-terminus (or C-terminus) relative to native HLA-A2 structures, except for minor differences in some side chain orientations within the A pocket^27^. It is also significant because, in native MHC I structures, the A and B pockets anchor the peptide terminal amino group and P2 residue, respectively, that contribute to protein stability. The FA and FV 20mer structures thus likely captured intermediate conformations adopted by MHC I when actively “evaluating” peptide P1 and P2 residues as part of editing, with the exact molecular features of MHC I intermediates expected to be fluid as maturation proceeds based on our MD simulations.

Previous studies indicated that molecular dynamics around the F pocket is a major driving force for recognition of MHC I molecules by tapasin and TAPBRP^7–15^. Conformational flexibility at the N-terminus of the groove of MHC II molecules is also a critical determinant of HLA-DM-mediated mechanism of peptide exchange^41^. As such, our structures of FA and FV 20mers thus raise the intriguing question of whether the region close to the A and B pockets represents a binding surface recognized by a protein, yet to be identified, with a role in stabilizing MHC I intermediates and/or peptide editing. It is interesting that the cryo-EM structure of the PLC showed that the long P-domain of CRT chaperone is positioned atop and across the MHC I groove with its tip interacting with ERp57^16^. In this spatial organization, the P-domain could interact transiently with the partially folded region of the A and B pockets, as facilitated by the inherent structural plasticity of the P-domain and dynamic nature of the P-domain/ERp57 interaction^56,57^. More work is required to examine this idea. The possibility that ERAP1, ERAP2, and/or ERAP1/ERAP2 heterodimer play a functional role at the N-terminal end of the groove is also very reasonable. We showed previously in biochemical studies that ERAP1 and ERAP1/ERAP2 can actively trim the protruding N-terminal residues of long peptides, including the AA 20mer, while bound to HLA-B8E76C^28,29^. Others also showed that ERAP1 trims peptides bound to H2-K^b^ using cell-based assays^53^. Moreover, it was demonstrated that mouse ERAAP (equivalent to ERAP1) synergizes with tapasin to edit peptide repertoires^58^. In fact, a role for MHC I molecules in antigen processing has long been suggested^59^. Our current study supports this view and furthermore suggests that the ERAP enzymes are more likely engaging with intermediate forms of MHC I molecules, rather than correctly conformed molecules^60^.

In conclusion, our study provided a crystallographic and MD characterization of conformational substates in MHC I-peptide systems, and it brought into focus the N-terminal end of the groove in mechanisms of high-affinity peptide selection. This understanding is critical given the role of MHC I-restricted peptide repertoires for activation of adaptive immune responses to control viral infections. Finally, our work opens new avenues to examine chaperoning of the groove around the A pocket, and it also encourages further characterization of other MHC I molecules.

## Methods

### Synthetic peptides.

Peptides were synthesized by the solid-phase methodology (GenScript Biotech Co.) and purified by reverse-phase chromatography on a C18 HPLC column. Stock solutions of peptides in DMSO were stored at −80°C.

### Refolding of HLA-B*0801E76C complexes.

Using the crystal structure of HLA-B*0801/GGRKKYKL (PDB code 1AGB), we identified residue Glu76 to be geometrically well-positioned to form a disulfide bond with the side chain of P7 peptide residue, after mutation with a cysteine, as we described previously^28^. The HLA-B*0801E76C heavy chain mutant was generated as described previously^28^. HLA-B*0801E76C complexes were reconstituted from urea-solubilized inclusion bodies of HLAB*0801E76C heavy chain (1 μM) and b_2_-microglobulin (2 μM) with a synthetic Cys-P7 peptide (10 μM) in an oxidative refolding buffer at 4°C^61^. After 48 hours, the crude refolding mixture of HLA-B*0801E76C complexes was purified on a Superdex-200 size exclusion chromatography column by FPLC. Stock solutions of purified complexes (10–30 mg/ml) in 20 mM Tris-HCl, pH 7.5, 150 mM NaCl, were kept at −80 °C.

### Crystallization.

The initial crystallization condition of HLA-B*0801E76C/(RA)_6_ FAKKKYCL (10 mg/ml) was identified using the Crystal Screen^™^ (Hampton Research, Riverside, CA) as solution #9 (0.2 M ammonium acetate, 0.1 M sodium citrate tribasic dihydrate, pH 5.6, 30% (w/v) PEG 4000) via the hanging-drop vapor diffusion method at room temperature. The initial crystals were optimized using different pH values (4.5–7.0) and PEGs (6000–10000; 10–30%). These optimized crystals were used to generate a seeding solution in solution #9. Crystals used for data collection were grown by mixing 2 μl of 10 mg/ml protein solution with 2 μl of 0.2 M ammonium acetate, 18% PEG 4000, 0.1 M sodium citrate, pH 5.7, and 0.5 μl of seeding solution. Similar crystallization conditions were used to collect data for HLA-B*0801E76C loaded with FA and FV 8mers and FV 20mer.

### Data collection, structural determination, and refinement.

X-ray diffraction data sets were collected with a MAR-225 CCD detector at the LS-CAT beamline 21-ID-G (or 21-ID-F) of the Advanced Photon Source (Argonne National Laboratory, Argonne, IL). Data were integrated and scaled with the HKL2000 program package^62^ or XDS^63^. Details of data processing are indicated in Supplementary Table 1. The structures of all complexes were solved by molecular replacement using Phaser^64^ (the initial search model was HLA-B*0801E76C/R(N-Me)AAAKKKYCL (PDB code 6P2S). Structure refinement of all models was carried out in Phenix (or Refmac in CCP4)^65–67^ and manual building with COOT^68^. Final refinement statistics are summarized in Supplementary Table 1. The atomic coordinates of all structures have been deposited in the Protein Data Bank with the following accession codes: FA 8mer (8E13), FA 20mer (8E2Z), FV 8mer (8E81), and FV 20mer (8EC5).

### Thermal denaturation assay.

A thermal denaturation assay was performed using reaction mixtures consisting of 7 μl of a complex (final concentration of 2 μM), 7 μl of 10x SYPRO orange dye (5000x, Thermo Fisher Scientific, Waltham, MA) and 7 μl of 50 mM HEPES, pH 7.2, 150 mM NaCl. Each mixture (total volume 21 μl) was analyzed in quadruplicate using an ABI ViiA7 RT-PCR instrument (Life Technologies, Inc., Carlsbad, CA). A temperature gradient from 25 to 95°C with continuous increment of 0.06°C/sec was used to generate the denaturation curves. The averaged denaturation curves were plotted as “fluorescence intensity” versus “temperature”, and the minimum point of the first derivative of each curve provided the melting temperature.

### MD initial structures preparation.

The x-ray crystal structures of HLA-B8E76C loaded with five different peptides were analyzed by MD simulations: FA and FV 8mers (this study), AA 20mer (6P2C)^29^, and FA and FV 20mers (this study). The missing regions of HLA-B8E76C in the structures of FA and FV 20mers were complemented using software UCSF Chimera^69^ with the structure of AA 20mer as the template. The missing regions of the bound 20mer peptides were complemented using Modeller^70^ integrated in software UCSF Chimera^69^. In the simulations, the peptide termini were neutralized to exclude artificial charge effects.

### MD simulations set-up.

Depending on the purpose, two different MD simulations were performed: replica exchange MD (REMD) and plain MD. The REMD simulations were used for the thermal properties analysis due to its efficiency in thermal equilibrated conformational ensemble sampling, while the plain MD simulations were used for simulating the rotation of the terminal amino group of bound FA20..8mer and FV20..8mer from their unusual up orientations. Both types of MD simulations included common settings, as follows. Simulations were performed with explicit solvent using the software package GROMACS 5.1.1^71,72^. The force field CHARMM36m^73^ together with its own modified TIP3P water model^74^ was also used. The LINCS algorithm was applied to constrain the covalent bonds with H-atoms, and the time step in simulation was 2 fs. The protein was simulated in 0.l M aqueous NaCl solution. After a short energy minimization, an NVT simulation of 100 ps with the V-rescale temperature coupling at 310 K was performed, followed by an NPT simulation of 300 ps with the Parrinello–Rahman coupling method at a reference pressure of 1 bar. The relaxation times for the temperature coupling and pressure coupling are 0.1 ps and 2 ps, respectively. During NVT and NPT simulations, the protein backbone is constrained to its initial structure. At the end, we removed the constraints and performed production simulations at the same temperature and pressure. The time interval for conformational sampling in simulations was 20 ps.

### REMD simulations.

After a short thermal equilibration process with NVT and NPT, as described above, we set up 30 replicas with temperature distributed from 300 K to 340 K following the webserver (https://virtualchemistry.org/remd-temperature-generator/)^75^ with an attempt swap duration of 1 ps between two neighboring temperatures. The average acceptance probability for the replica exchanges was about 30%. Each replica ran for 40 ns, and thus we have a simulation with total time up to 1.2 μs. The thermal equilibrated ensembles were collected from the replica at temperature 310 K from the REMD simulations, from which the flexibility of HLA-B8E76C heavy chain and interactions between peptide residue P1 and MHC I residues Tyr59 and Ile52 were analyzed.

### Plain MD simulations.

Using the FA and FV 20mer structures, as generated in the “Initial structures preparation” stage, we generated the initial configurations of FA20..8mer and FV20..8mer by deleting the extension (RA)_6_ residues. After a short thermal equilibration process with NVT and NPT as described above, we performed the production run for 300 ns to simulate the relaxation process of FA20..8mer and FV20..8mer. During the simulations, we observed rotations of the terminal amino groups in FA20..8mer and FV20..8mer peptides. We repeated the simulation three times for each system to ensure producibility.

### Interaction Analysis.

We identified possible interactions between peptide residue P1 and MHC I residues Tyr59 and Ile52 according to the geometrical properties, i.e., distances and relative orientations between the two residues. For interaction between P1 Phe and Tyr59, the distance was defined between the geometrical centers of the two aromatic rings, while the relative orientation was defined by the angle between the normal directions of the aromatic rings. For interaction between P1 Ala of AA 20mer and Tyr59, the distance was defined between the Cβ atom of P1 Ala and the geometrical center of the aromatic ring of Tyr59. For interaction between P1 Phe and Ile52, the distance was defined between the geometrical center of the aromatic ring in P1 Phe and the Cδ atom of Ile 52, while the angle C-H-X was defined by the atoms Cδ of Ile52, hydrogen atom covalently bonded to Cδ and the center of the Phe aromatic ring. Finally, for interaction between P1 Ala of AA 20mer and Ile52, the distance was defined between the Cβ atom in P1 Ala and the Cδ atom of Ile52.

### Analysis of rotations of peptide terminal amino groups.

We probed rotations of terminal amino groups in pocket A by monitoring the evolution of the dihedral angle ω formed by atoms Cys76:CA-P7:CA-P1:CA-P1:N (Supplementary Fig. 6A) in the plain MD simulations. When drawing evolution curves, we reasonably required that the rotation between two neighboring frames be less than 180°, i.e., |*ω*(*t*_*i*+1_) − *ω*(*t*_*i*_)| < 180°. Otherwise, we added/subtracted the dihedral angle *ω*(*t*_*i*+1_) by 360°. In the end, to eliminate thermal fluctuations and visualize the rotation pathways better, we smoothed the evolution curves by an average sliding window of 10 ns.

## References

[1] Saper M. A., Bjorkman P. J. & Wiley D. C. Refined structure of the human histocompatibility antigen HLA-A2 at 2.6 Å resolution. J. Mol. Biol. 219, 277–319 (1991). 2038058 10.1016/0022-2836(91)90567-p

[2] Matsumura M., Fremont D. H., Peterson P. A. & Wilson I. A. Emerging principles for the recognition of peptide antigens by MHC class I molecules. Science 257, 927–934 (1992). 1323878 10.1126/science.1323878

[3] Fahnestock M. L., Johnson J. L., Feldman R. M., Tsomides T. J., Mayer J., Nahri L. O. & Bjorkman P. J. Effects of peptide length and composition on binding to an empty class I MHC heterodimer. Biochem. 33, 8149–8158 (1994). 8025120 10.1021/bi00192a020

[4] Madden D. R. The three-dimensional structure of peptide-MHC-complexes. Annu. Rev. Immunol. 13, 587–622 (1995). 7612235 10.1146/annurev.iy.13.040195.003103

[5] Bouvier M. & Wiley D. C. Importance of peptide amino and carboxyl termini to the stability of MHC class I molecules. Science 265, 398–402 (1994). 8023162 10.1126/science.8023162

[6] Cresswell P., Bangia N., Dick T & Diedrich G. The nature of the MHC class I peptide loading complex. Immunol. Rev. 171, 21–28 (1999). 10.1111/j.1600-065x.1999.tb01353.x 10631934

[7] Van Hateren A., Bailey A., Werner J. M. & Elliott T. Plasticity of empty major histocompatibility complex molecules determines peptide-selector function. Molec. Immunol. 68, 98–101 (2015). 25818313 10.1016/j.molimm.2015.03.010 PMC4726658

[8] Ayres C. M., Corcelli S. A. & Baker B. M. peptide and peptide-dependent motions in MHC proteins: immunological and biophysical underpinnings. Front. Immunol. 8, 935 (2017). 28824655 10.3389/fimmu.2017.00935 PMC5545744

[9] Yanaka S. & Sugase K. Exploration of the conformational dynamics of major histocompatibility complex molecules. Frontiers Immunol. 8, 632 (2017). 10.3389/fimmu.2017.00632 PMC5446982 28611781

[10] Wieczorek M. Abualrous E. T., Stich J., Alvaro-Benito M., Stolzenberg S., Noe F. & Freund C. Major histocompatibility complex (MHC) class I and MHC class II proteins: conformational plasticity in antigen presentation. Front. Immunol. 8, 292 (2017). 28367149 10.3389/fimmu.2017.00292 PMC5355494

[11] Fisette O., Wingbermuhle, Tampe R. & Schafer L.V. Molecular mechanism of peptide editing in the tapasin-MHC I complex. Sc. Reports 19085 (2016). 10.1038/srep19085 PMC4709564 26754481

[12] Truong H. V. & Sgourakis N. G. Dynamics of MHC I-molecules in the antigen processing and presentation pathway. Curr. Opin. Immunol. 70, 122–128 (2021). 34153556 10.1016/j.coi.2021.04.012 PMC8622473

[13] Jantz-Naeem N. & Springer S. Venus flytrap or pas de trois? The dynamics of MHC class I molecules. Curr. Opin. Immunol. 70, 82–89 (2021). 33993034 10.1016/j.coi.2021.04.004

[14] Thomas C. & Tampe R. MHC I assembly and peptide editing – chaperones, clients, and molecular plasticity in immunity. Curr. Opin. Immunol. 70, 48–56 (2021). 33689959 10.1016/j.coi.2021.02.004

[15] Margulies D. H., Taylor D. K., Jiang J., Boyd L. F., Ahmad J., Mage M. G. & Natarajan K. Chaperones and catalysts: How antigen presentation pathways cope with biological necessity. Front. Immunol. 13, 859782 (2022). 35464465 10.3389/fimmu.2022.859782 PMC9022212

[16] Blees A., Januliene D., Hofmann T., Koller N., Schmidt C., Trowitzsch S., Moeller A. & Tampe R. Structure of the human MHC I peptide-loading complex. Nature 551, 525–528 (2017). 29107940 10.1038/nature24627

[17] Chen M. & Bouvier M. Analysis of interaction in a tapasin/class I complex provides a mechanism for peptide selection. EMBO J. 26, 1681–1690 (2007). 17332746 10.1038/sj.emboj.7601624 PMC1829385

[18] Herman C., Strittmatter L. M., Deanne J. E. & Boyle L. H. The binding of TAPBPR and tapasin to MHC class I is mutually exclusive. J. Immunol. 191, 5743–5750 (2013). 24163410 10.4049/jimmunol.1300929 PMC3836178

[19] Herman C., van Hateren A., Trautwein N., Neerincx A., Duriez P. J., Stevanovic S., Trownsdale J., Deane J. E., Elliott T. & Boyle L. H. TAPBPR alters MHC class I peptide presentation by functioning as a peptide catalyst. eLife 4, e09617 (2015). 26439010 10.7554/eLife.09617 PMC4718805

[20] Thomas C. & Tampe R. Structure of the TAPBPR-MHC I complex defines the mechanism of peptide loading and editing. Science 358, 1060–1064 (2017). 29025996 10.1126/science.aao6001

[21] Jiang J., Natarajan K., Boyd L. F., Morozov G. I., Mage M. G. & Margulies D. H. Crystal structure of the TAPBPR-MHC I complex reveals the mechanism of peptide editing in antigen presentation. Science 358, 1064–1068 (2017). 29025991 10.1126/science.aao5154 PMC6320693

[22] McShan A., Devlin C. A., Overall S. A., Park S. A., Toor J. S., Moschidi D., Flores-Solis D., Choi H., Tripathi S., Procko S. & Sgourakis N. G. Molecular determinants of chaperone interactions on MHC-I folding and antigen repertoire selection. Proc. Natl. Acad. Sci. USA 116, 25602–25613 (2019). 31796585 10.1073/pnas.1915562116 PMC6926029

[23] Van Hateren A., Anderson M., Bailey A., Werner J. M. Skipp P. & Elliott T. Direct evidence for conformational dynamics in major histocompatibility complex class I molecules. J. Biol. Chem. 292, 20255–20269 (2017). 29021251 10.1074/jbc.M117.809624 PMC5724011

[24] Bouvier M. & Wiley D. C. Structural characterization of a soluble and partial folded class I major histocompatibility heavy chain/β_2_m heterodimer. Nat. Struct. Biol. 5, 377–384 (1998). 9587000 10.1038/nsb0598-377

[25] Fahnestock M. L., Tamir I., Narhi L. & Bjorkman P. J. Thermal stability comparisons of purified empty and peptide-filled forms of a class I MHC molecule. Science 258, 1658–1662 (1992). 1360705 10.1126/science.1360705

[26] Kurimoto E., Kuroki K., Yamaguchi Y., Yagi-Utsumi M., Igaki T., Iguchi T., Maenaka K. & Kato K. Structural and functional mosaic nature of MHC class I molecules in their peptide-free form. Molec. Immunol. 55, 393–399 (2013). 23578712 10.1016/j.molimm.2013.03.014

[27] Anjanappa R., Garcia-Alai M., Kopicki J.-D., Lockhauserbaumer J., Aboelmagd M., Hinrichs J., Nemtanu I. M., Uetrecht C., Zacharias M., Springer S. & Meijers R. Structures of peptide-free and partially loaded MHC class I molecules reveal mechanisms of peptide selection. Nat. Comm. 11, 1314 (2020). 10.1038/s41467-020-14862-4 PMC7066147 32161266

[28] Chen H., Li L, Weimershaus M., Evnouchidou I., van Endert P. & Bouvier M. ERAP1-ERAP2 dimers trim MHC I-bound precursor peptides; implications for understanding peptide editing. Sc. Rep. 6, 28902 (2016). 27514473 10.1038/srep28902 PMC4981824

[29] Li L., Batliwala M. & Bouvier M. ERAP1 enzyme-mediated trimming and structural analyses of MHC I-bound precursor peptides yield novel insights into antigen processing and presentation. J. Biol. Chem. 294, 18534–18544 (2019). 31601650 10.1074/jbc.RA119.010102 PMC6901306

[30] Phillips R. E., Rowland-Jones S., Nixon D. F., Gotch F. M., Edwards J. P., Ongulesi A. O., Elvin J. G., Rothbard J. A., Bangham C. R., Rizza C. R. & McMichael A. J. Human immunodeficiency virus genetic variation that can escape cytotoxic T cell recognition. Nature 354, 453–459 (1991). 1721107 10.1038/354453a0

[31] Tehrani A. Z. & Kim K. S. Functional molecules and materials by π-interaction based quantum theoretical design. Int. J. Quantum Chem. 116, 622–633 (2016).

[32] Wang J. & Yao L. Dissecting C-H⋯π and N-H⋯π interactions in two proteins using a combined experimental and computational approach. Sci. Rep. 9, 20149 (2019). 31882834 10.1038/s41598-019-56607-4 PMC6934659

[33] Ng J. H. J., Tachedjian M., Deakin J., Wynne J. W., Cui J., Haring V., Broz I., Chen H., Belov K., Wang L.-F. & Baker M. L. Evolution and comparative analysis of the bat MHC -I region. Sc. Rep. 6, 21256 (2016). 26876644 10.1038/srep21256 PMC4753418

[34] Lu D., Liu K., Zhang D., Yue C., Lu Q., Cheng H., Wang L., Chai Y., Qi J., Wang L.-F., Gao G. F. & Liu W. J. Peptide presentation by bat MHC class I provides new insights into the antiviral immunity of bats. PLos Biol. 9, e30000436 (2019). 10.1371/journal.pbio.3000436 PMC6752855 31498797

[35] Qu Z., Li Z., Ma L., Wei X., Zhang L., Liang R., Meng G., Zhang N. & Xia C. Structure and peptidome of the bat MHC class I molecule reveal a novel mechanism leading to high-affinity peptide binding. J. Immunol. 202, 3493–3506 (2019). 31076531 10.4049/jimmunol.1900001 PMC6545463

[36] Natarajan S. K., Stern L. J. & Sadegh-Nasseri S. Sodium dodecyl sulfate stability of HLA-DR1 complexes correlates with burial of hydrophobic residues in pocket 1. J. Immunol. 162, 3463–3470 (1999). 10092802

[37] Zarutskie J. A., Sato A. K., Rusche M. M., Chan C., Lomakin A., Benedek G. B. & Stern L. J. A conformational change in the human major histocompatibility complex protein HLA-DR1 induced by peptide binding. Biochem. 38, 5878–5887 (1999). 10231540 10.1021/bi983048m

[38] Sato A. K., Zarutskie J. A., Rusche M. M., Lomakin A., Natarajan S. K., Sadegh-Nasseri S., Benedek G. B. & Stern L. J. Determinants of the peptide-induced conformational change in the human class II major histocompatibility complex protein HLA-DR1. J. Biol. Chem. 275, 2165–2173 (2000). 10636922 10.1074/jbc.275.3.2165

[39] Chou C. L. & Sadegh-Nasseri S. HLA-DM recognized the flexible conformation of major histocompatibility complex class II. J. Expm. Med. 192, 1697–1706 (2000). 10.1084/jem.192.12.1697 PMC2213500 11120767

[40] Pos W., Sethi D. K., Call M. J., Schulze M.-S. E. D., Anders A.-K., Pyrdol J. & Wucherpfennig K. W. Crystal structure of the HLA-DM-HLA-DR1 complex defines mechanism for rapid peptide selection. Cell 151, 1557–1568 (2012). 23260142 10.1016/j.cell.2012.11.025 PMC3530167

[41] Painter C. A., Negroni M. P., Kellesberger K. A., Zavala-Ruiz Z., Evans J. E. & Stern L. J. Conformational lability in the class II MHC 3_10_ helix and adjacent extended strand dictate HLA-DM susceptibility and peptide exchange. Proc. Natl. Acad. Sci. USA 108, 19329–19334 (2011). 22084083 10.1073/pnas.1108074108 PMC3228433

[42] Yanaka S., Ueno T., Shi Y., Qi J., Gao G. F., Tsumoto K. & Sugase K. Peptide-dependent conformational fluctuation determines the stability of the human leukocyte antigen class I complex. J. Biol. Chem. 289, 24680–24690 (2014). 25028510 10.1074/jbc.M114.566174 PMC4148890

[43] Hawse W., Gloor B. E., Ayres A. M., Kho K., Nuter E. & Baker B. M. Peptide modulation of class I major histocompatibility complex protein molecular flexibility and the implications for immune recognition. J. Biol. Chem. 288, 24372–24381 (2013). 23836912 10.1074/jbc.M113.490664 PMC3750139

[44] Flad T., Spengler B., Kalbacher H., Brossart P., Baier D., Kaufmann R., Bold P., Metzger S., Bluggel M., Meyer H. E., Kurz B. & Muller C. A. Direct identification of major histocompatibility complex class I-bound tumor-associated peptide antigens of a renal carcinoma cell line by a novel mass spectrometric method. Cancer Res. 58, 5803–5811 (1998). 9865739

[45] Stickel J. S., Weinzierl A. O., Hillen O., Drews O., Schuler M. M., Hennenlotter J., Wernet D., Muller C. A., Stenzl A., Rammensee H.-G. & Stevanovic S. HLA- ligand profiles of primary renal cell carcinoma maintained in metastases. Cancer Immunol. Immunother. 58, 1407–1417 (2009). 19184600 10.1007/s00262-008-0655-6 PMC11031011

[46] Adair S. J., Carr T. M., Fink M. J., Slingluff C. L. Jr & Hogan K. T. The TAG family of cancer/testis antigens is widely expressed in a variety of malignancies and gives rise to HLA-A2-restricted epitopes. J. Immunother. 31, 7–17 (2008). 18157007 10.1097/CJI.0b013e318159f797

[47] Koziel M. J., Dudley D., Afdhal N., Grakuoi A., Rice C. M., Choo Q. L., Houghton M. & Walker B. D. HLA class I-restricted cytotoxic T lymphocytes specific for hepatitis C virus. Identification of multiple epitopes and characterization of patterns of cytokine release. J. Clin. Invest. 96, 2311–2321 (1995). 7593618 10.1172/JCI118287 PMC185882

[48] Burrows S. R., Sculley T. B., Misko I. S., Schmidt C. & Moss D. J. An Epstein-Barr virus-specific cytotoxic T cell epitope in EBV nuclear antigen 3 (EBNA 3). J. Exp. Med. 171, 345–349 (1990). 1688611 10.1084/jem.171.1.345 PMC2187672

[49] Steven N. M., Leese A. M., Annels N. E., Lee S. P. & Rickinson A. B. epitope focusing in the primary cytotoxic T cell response to Epstein-Barr virus and its relationship to T cell memory. J. Expm. Med. 184, 1801–1813 (1996). 10.1084/jem.184.5.1801 PMC2192864 8920868

[50] Hansen T. H., Lybarger L., Yu L., Mitaksov V. & Fremont D. H. Recognition of open conformers of classical MHC by chaperones and monoclonal antibodies. Immunol. Rev. 207, 100–111 (2005). 16181330 10.1111/j.0105-2896.2005.00315.x

[51] Mage M. G., Dolan M. A., Wang R., Boyd L. F. Revilleza M. J., Robinson, Natarajan K., Myers N. B., Hansen T. H. & Margulies D. H. The peptide-receptive transition state of MHC class I molecules: insights from structures and molecular dynamics. J. Immunol. 189, 1391–1399 (2012). 22753930 10.4049/jimmunol.1200831 PMC3422668

[52] Mage M. G., Dolan M. A., Wang R., Boyd L. F. Revilleza M. J., Robinson, Natarajan K., Myers N. B., Hansen T. H. & Margulies D. H. A structural and molecular dynamics approach to understanding the peptide-receptive transition state od MHC-I molecules. Molec. Immunol. 55, 123–125 (2013). 23200143 10.1016/j.molimm.2012.10.021 PMC3632263

[53] Papakyriakou A., Reeves E., Beton H., Mikolajek H., Douglas L., Cooper G., Elliott T., Werner J. M. & James E. The partial dissociation of MHC class I-bound peptides exposes their N terminus to trimming by endoplasmic reticulum aminopeptidase 1. J. Biol. Chem. 293, 7538–7648 (2018). 29599287 10.1074/jbc.RA117.000313 PMC5961055

[54] Fisette O., Wingbermuhle, & Schafer L.V. Partial dissociation of truncated peptide influences the structural dynamics of the MHC I binding groove. Front. Immunol. 8, 408 (2017). 28458665 10.3389/fimmu.2017.00408 PMC5394104

[55] Wingbermuhle S. & Schafer L. V. Partial peptide dissociation and binding groove plasticity in two major histocompatibility complex class I alleles – differences between alleles versus force field and sampling effects. RSC Adv. 12, 29908–29914 (2022). 36321080 10.1039/d2ra05324a PMC9580618

[56] Ellgaard L., McNaul N., Chatsisvili A., Braakman I. Co- and post-translational protein folding in the ER. Traffic 17, 615–638 (2016). 26947578 10.1111/tra.12392

[57] Fisette O. Schroder G. F. & Schafer L. V. Atomistic structure and dynamics of the human MHC-I peptide-loading complex. Proc. Natl. Acad. Sci. USA 117, 20597–20606 (2020). 32788370 10.1073/pnas.2004445117 PMC7456110

[58] Kanaseki T., Blanchard N., Hammer G. E., Gonzalez F. & Shastri N. ERAPP synergizes with MHC class I molecules to make the final cut in the antigenic peptide precursors in the endoplasmic reticulum. Immunity 25, 795–806 (2006). 17088086 10.1016/j.immuni.2006.09.012 PMC2746443

[59] Falk K., Rotzschke O. & Rammensee H. G. Cellular peptide composition governed by major histocompatibility complex class I molecules. Nature 348, 248–251 (1990). 2234092 10.1038/348248a0

[60] Mavridis G., Arya R., Domnick A., Zoidakis J., Makridakis M., Vlahou A., Mpakali A., Lelis A., Georgiadis D., Tampe R., Papakyriakou A., Stern L. J. & Stratikos E. A systematic re-examination of processing of MHC I-bound antigenic peptide precursors by endoplasmic reticulum aminopeptidase 1. J. Biol. Chem. 295, 7193–7210 (2020). 32184355 10.1074/jbc.RA120.012976 PMC7247305

[61] Garboczi D. N., Hung D. T. & Wiley D. C. HLA-A2-peptide complexes: refolding and crystallization of molecules expressed in Escherichia coli and complexed with single antigenic peptides. Proc. Natl. Acad. Sci. U.S.A. 89, 3429–3433 (1992). 1565634 10.1073/pnas.89.8.3429 PMC48881

[62] Otwinowski Z. & Minor W. Processing of X-ray diffraction data collected in oscillation mode. Methods Enzymol. 276, 307–326 (1997). 27754618 10.1016/S0076-6879(97)76066-X

[63] Kabsch W. XDS. Acta Crystallogr. D Biol. Crystallogr. 66, 125–132 (2010). 20124692 10.1107/S0907444909047337 PMC2815665

[64] McCoy A. J., Grosse-Kunstleve R. W., Adams P. D., Winn M. D., Storoni L. C. & Read R. J. Phaser crystallographic software. J. Appl. Crystallogr. 40, 658–674 (2007). 19461840 10.1107/S0021889807021206 PMC2483472

[65] Adams P. D., Afonine P. V., Bunkoczi G., Chen V. B., Davis I. W., Echols N., Headd J. J., Hung L. W., Kapral G. J., Grosse-Kunstleve R. W., McCoy A. J., Moriarty N. W., Oeffner R., Read R. J., Richardson D. C., PHENIX: a comprehensive Python-based system for macromolecular structure solution. Acta Crystallogr. D Biol. Crystallogr. 66, 213–221 (2010). 20124702 10.1107/S0907444909052925 PMC2815670

[66] Winn M. D., Murshudov G. N. & Papiz M. Z. Macromolecular TLS refinement in REFMAC at moderate resolutions. Methods Enzymol. 374, 300–321 (2003). 14696379 10.1016/S0076-6879(03)74014-2

[67] Winn M. D., Ballard C. C., Cowtan K. D., Dodson E. J., Emsley P., Evans P. R., Keegan R. M., Krissinel E. B., Leslie A. G., McCoy A., McNicholas S. J., Murshudov G. N., Pannu N. S., Potterton E. A., Powell H. R., Read R. J., Vagin A. & Wilson K. S. Overview of the CCP4 suite and current developments. Acta Crystallogr. D Biol. Crystallogr. 67, 235–242 (2011). 21460441 10.1107/S0907444910045749 PMC3069738

[68] Emsley P. & Cowtan K. Coot: model-building tools for molecular graphics. Acta Crystallogr. D Biol. Crystallogr. 60, 2126–2132 (2004). 15572765 10.1107/S0907444904019158

[69] Pettersen E. F., Goddard T. D., Huang C. C., Couch G. S., Greenblatt D. M., Meng E. C. & Ferrin T. E. UCSF Chimera--a visualization system for exploratory research and analysis. J. Comput. Chem. 25, 1605–1612 (2004). 15264254 10.1002/jcc.20084

[70] Sali A., & Blundell T. L. Comparative protein modelling by satisfaction of spatial restraints. J. Mol. Biol. 234, 779–815 (1993). 8254673 10.1006/jmbi.1993.1626

[71] Pronk S., Pall S., Schulz R., Larsson P., Bjelkmar P., Apostolov R., Shirts M. R., Smith J. C., Kasson P. M., van der Spoel D., Hess B. & Lindahl E. GROMACS 4.5: A high-throughput and highly parallel open source molecular simulation toolkit. Bioinformatics 29, 845–85 (2013). 23407358 10.1093/bioinformatics/btt055 PMC3605599

[72] Abraham M. J., Murtola T., Schulz R., Pall S., Smith J. C., Hess B. & Lindahl E. GROMACS: High performance molecular simulations through multi-level parallelism from laptops to supercomputers. SoftwareX 1−2, 19–25 (2015).

[73] Huang J., Sarah R., Grzegorz N., Ting R., Michael F., de Groot B. L., Helmut G. & MacKerell A. D. Jr. CHARMM36m: an improved force field for folded and intrinsically disordered proteins, Nat. Methods 14, 71–76 (2017). 27819658 10.1038/nmeth.4067 PMC5199616

[74] Mark P. & Nilsson L. Structure and dynamics of liquid water with different long‐range interaction truncation and temperature control methods in molecular dynamics simulations. J. Comput. Chem. 23, 1211–1219 (2002). 12210146 10.1002/jcc.10117

[75] Patriksson A. & van der Spoel D. A temperature predictor for parallel tempering simulations Phys. Chem. Chem. Phys. 10, 2073–2077 (2008). 18688361 10.1039/b716554d

